# Supplementary material for: The Mediating Role of Psychological Balance on the Effects of Dietary Behavior on Cognitive Impairment in Chinese Elderly
Source: Nutrients. 2024 Mar 21;16(6):908. doi: 10.3390/nu16060908 (PMC10974113; doi:10.3390/nu16060908)
Supplement: Supplementary file 1 [file nutrients-16-00908-s001.zip › nutrients-2880981-supplementary.pdf]

Supplementary Material

Table S1. The response probabilities of seven food groups in four dietary patterns in the subjects (n, %).

Cluster	Vegetables	Fruits	Red Meat	Fish	Eggs	Beans-nuts	Milk
DP 1	2896 (85.63)	915 (27.05)	2044 (60.44)	639 (18.89)	0 (0.00)	546 (16.14)	280 (8.27)
DP 2	2383 (81.64)	951 (32.58)	1353 (46.35)	0 (0.00)	2765 (94.72)	905 (31.00)	1074 (36.79)
DP 3	2937 (87.59)	0 (0.00)	3159 (84.21)	2529 (75.42)	3137 (93.56)	2488 (74.20)	1246 (37.16)
DP 4	4586 (98.33)	4664 (100.00)	4449 (95.39)	3598 (77.14)	4418 (94.73)	3426 (73.46)	2990 (64.11)
Total	12802 (89.41)	6530 (45.61)	11005 (76.86)	6766 (47.26)	10320 (72.08)	7365 (51.44)	5590 (39.04)
